# Isotopic Niche of Three Sympatric Mustelids

**DOI:** 10.3390/life16020208

**Published:** 2026-01-27

**Authors:** Linas Balčiauskas, Andrius Garbaras, Rasa Vaitkevičiūtė Koklevičienė, Inga Garbarienė, Laima Balčiauskienė

**Affiliations:** 1 State Scientific Research Institute Nature Research Centre, LT-08412 Vilnius, Lithuania; laima.balciauskiene@gamtc.lt; 2 Center for Physical Sciences and Technology, Saulėtekio Ave. 3, LT-10257 Vilnius, Lithuania; andrius.garbaras@ftmc.lt (A.G.); inga.garbariene@ftmc.lt (I.G.); 3 Agriculture Academy, Vytautas Magnus University, Studentų 11, Akademija, LT-53361 Kaunas, Lithuania; rasa.vaitkeviciute1@vdu.lt; 4 VšĮ Forest 4.0, Akademija, LT-53361 Kaunas, Lithuania

**Keywords:** *Martes martes*, *Martes foina*, *Mustela putorius*, isotopic niche, diet, trophic overlap, synanthropy, mesocarnivores

## Abstract

Although sympatric carnivores typically exhibit dietary differentiation to reduce interspecific competition, contemporary isotopic comparisons of European mustelids remain scarce. In this study, we present the first modern stable isotope analysis of hair to evaluate the dietary niches and trophic relationships of pine martens (*Martes martes*), stone martens (*Martes foina*), and European polecats (*Mustela putorius*) in Lithuania and Latvia. The stable carbon and nitrogen isotope values differed among the species. *M. martes* showed lower δ^15^N values and more depleted δ^13^C signatures than the two more synanthropic species. Isotopic niche analyses revealed that *M. martes* occupied the broadest niche, while *M. foina* and *M. putorius* exhibited narrower niches with substantial overlap. Habitat influenced trophic position: individuals from settlements showed higher δ^15^N values than those from forests or wetlands. In contrast, sex- and age-related differences were weak or absent. These results demonstrate that despite partial spatial coexistence, sympatric mustelids differ primarily in isotopic niche structure rather than mean isotope values and that human-modified environments promote trophic convergence among generalist mesocarnivores. However, the small sample sizes for *M. foina* and *M. putorius* mean that estimates of isotopic niche width and overlap should be regarded as preliminary, and observed sex- and age-related patterns likely reflect limited statistical power rather than the absence of intraspecific dietary variation.

## 1. Introduction

The European pine marten (*Martes martes*) and the stone marten (*M. foina*) are closely related mustelids that often live in the same area. This coexistence is largely enabled by habitat and ecological niche partitioning [1]. *M. martes* is a forest specialist that relies on arboreal resting sites and woodlands for foraging [2], while *M. foina* is a habitat generalist that is strongly associated with rural and suburban environments [3]. Studies consistently show spatial segregation, with *M. martes* using forest interiors and *M. foina* exploiting human-modified landscapes [4,5], and differences in diet reflect these habitat affinities. *M. martes* primarily consume rodents, birds, and seasonal fruits [6,7,8,9,10], whereas *M. foina* shift toward fruits, invertebrates, and synanthropic prey, particularly in urban and Mediterranean habitats [11,12,13,14,15]. Frugivory in *M. foina* is more pronounced [16,17,18].

The European polecat (*Mustela putorius*) is classified as Least Concern globally [19], but E. Croose et al. [20] highlight growing evidence that the species is declining across much of its European range. Because the species is poorly monitored, confirming trends is difficult. It has a flexible diet consisting mainly of vertebrates, such as rodents and amphibians. Geographic variation ranges from populations that specialize in anurans to populations that depend on rabbits [21,22]. *M. putorius* and *M. foina* co-occur widely, but the two species differ in trophic emphasis, with *M. putorius* relying more heavily on vertebrate prey [23]. Despite occasional dietary overlap [24], competition between the two species is generally considered limited [25].

Decades of scat and stomach-content analysis documented the dietary patterns of mustelids across Europe [6,7,9,10,13,15,17,26,27,28]. Most of these studies rely on morphological methods, with relatively few incorporating molecular approaches, which leaves room for further refinement in our understanding of resource partitioning and ecological roles [29,30,31]. To date, no isotopic studies of these mustelid diets in Europe have been conducted.

Throughout much of its range, *M. putorius* co-occurs with *M. foina*, a more omnivorous mesocarnivore [23,25]. While their diets overlap, *M. putorius* typically rely on amphibians and small mammals, whereas *M. foina* consume more fruits, invertebrates, and synanthropic rodents, such as Norway rats (*Rattus norvegicus*) [23]. Diet overlap increases in winter and in agricultural landscapes with abundant rodents [23,24]. While competition is generally considered limited [21,22,25], the recent expansion of *M. foina* has renewed interest in their trophic interactions. No recent isotopic studies of *M. putorius* diets in Europe have been conducted.

In Lithuania, *M. martes* has an opportunistic diet with clear seasonal shifts. During the warm season, they consume a wide variety of foods, including small mammals (mainly rodents, such as *Clethrionomys glareolus*, *Microtus* voles, and *Apodemus* mice), birds, and plant material, as well as frequent but low-biomass insects [32,33]. In contrast, their winter diet consists primarily of mammals, which contribute more than 80–90% of total biomass. Rodents remain important, but ungulate carrion (e.g., wild boar and cattle) often dominates. Overall, *M. martes* in Lithuania exploit diverse prey in the summer while relying heavily on rodents and carrion in the winter [32,33]. The diets of *M. foina* and *M. putorius* in Lithuania have not been investigated.

The only reference for the isotopic niche of these European mustelid species is that of Selva et al. [34]. Their sample consists of 20 individuals collected between 1959 and 1962 representing two species of mustelids: *M. putorius*, with 14 specimens, and *M. martes*, with six specimens. The substantial temporal gap means that these historical samples cannot be directly compared with current isotopic niches of mustelid species.

According to Hobson, stable isotopes are an integrative tool for assessing diet assimilation, trophic relationships, and ecological niche differences [35]. This makes them ideal for evaluating interspecific, sex-based, and human-related dietary variation in mustelids. Metabolically inert tissues like hair preserve time-specific dietary signals, allowing us to understand how wildlife responds to environmental and anthropogenic changes.

We applied isotopic analysis to hair samples from three species of mustelids, *Martes martes*, *M. foina* and *Mustela putorius*, from Lithuania and Latvia to test for differences between them. We used hair, a metabolically inert tissue, because it preserves an individual’s dietary isotopic record [36,37]. Our aims were threefold: (1) compare isotopic space as a proxy for dietary space between these sympatric mustelid species to assess potential dietary differences, (2) examine intraspecific sex-based differences in stable isotope values within each species, and (3) test whether living closer to humans influences mustelid diets.

## 2. Materials and Methods

### 2.1. Study Site

**Figure 1:**
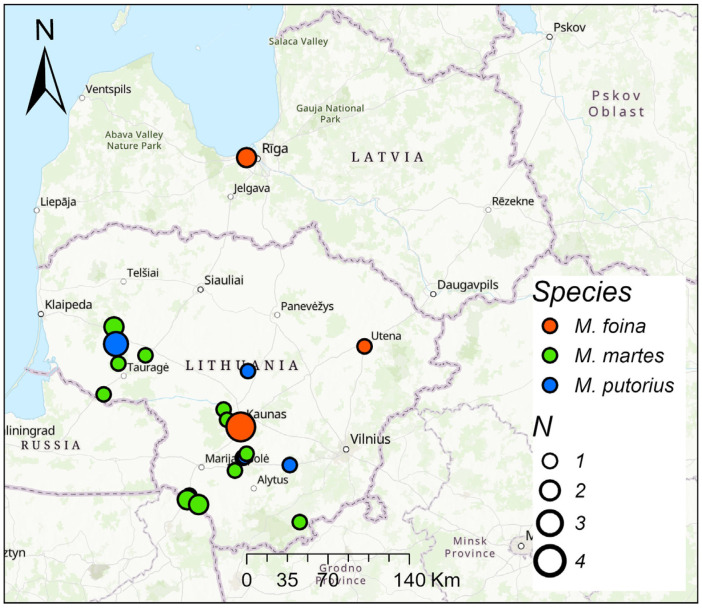
Sample collection sites of mustelids in Lithuania and Latvia. N is the number of colected individuals from the site.

From 2021 to 2025, the hair of *M. foina*, *M. martes,* and *M. putorius* was collected from legally hunted animals in Lithuania and a few in neighboring Latvia, from a total of 16 sites (Figure 1).

Mustelids were hunted in three habitats: forests, wetlands, and settlements, mainly rural ones. All *M. foina* were found in or near settlements. Of the seven *M. putorius*, six were found in settlements and one in a forest. *M. martes* were primarily hunted in forest habitats (N = 12), but also in wetlands and settlements (N = 4 in each).

Ethical review and approval were waived for this study because hair of mustelids was collected from legally hunted or roadkilled individuals. The authors themselves did not participate in the hunt. Mustelids were hunted in accordance with national and institutional guidelines from licensed third parties. This study did not involve the purposeful hunting of animals.

### 2.2. Sample Composition

Most of the individuals sampled were hunted during the 2023/2024 and 2024/2025 hunting seasons. One *M. martes* was sampled in 2021. In total, we analyzed 32 *M. martes*, six *M. foina*, and eight *M. putorius* from Lithuania. The sample from Latvia consisted of one *M. martes* and two *M. foina*. The breakdown of the Lithuanian sample is presented in Table S1.

The sex of the individuals was determined through dissection by the presence of testes or a uterus. However, because some hair samples were collected directly in the field, the sex and age of some mustelids could not be identified.

The age was determined based on cranial and skeletal features, particularly the morphology of the sagittal crest and the penis bone, baculum [38]. In young martens, the sagittal crest is barely visible, and the temporal ridges remain parallel. As the animals mature, the ridges in juveniles form a U- or V-shape, whereas in adults, they converge with the sagittal crest to form a Y-shape that becomes more pronounced with age [38,39]. However, comparisons with cementum annuli counts suggest that sagittal crest development varies among individuals, making it less reliable for precise aging, especially in juveniles [40].

Baculum characteristics (length, curvature, thickness, and number of proximal projections) also change predictably with age. Young specimens have a slender baculum with midshaft curvature; juveniles and adults, however, show more distal curvature, increased thickness, and greater length. Adults typically display multiple proximal projections compared to none or one in younger animals [39]. Morphometric analyses can distinguish between juveniles and adults with high accuracy [41].

We determined age using three criteria: baculum size and shape, sagittal crest form, and degree of tooth wear in the lower jaw [42]. All mustelids were assigned to one of three age classes: first year, second year, or older than two years.

### 2.3. Isotopic Analysis

The hair was collected either directly at the hunting site or later during animal dissection in the laboratory. A 4–5 mm wide tuft of hair was clipped from the shoulders, neck, and, in a few cases, the legs. Only fully grown guard hairs were sampled, and no actively growing (anagen) hairs were included. The collected hair was stored in plastic bags in a freezer at a temperature of −20 °C.

The sample preparation procedure involved transferring the hair samples to test tubes and rinsing them three times with deionized water while sonicating to remove surface debris. Then, the samples were immersed in a 2:1 methanol-to-chloroform solution for 10 min, followed by three additional rinses with deionized water. This process was repeated with a two-hour soaking period to ensure the thorough removal of potential contaminants. Finally, the samples were freeze-dried for 48 h and sealed in tin capsules for stable isotope analysis. Approximately 1 mg of hair was required for analyzing stable nitrogen and carbon isotopes.

The stable carbon and nitrogen isotopic compositions were determined using a Thermo Delta V continuous flow isotope ratio mass spectrometer (Thermo, Bremen, Germany) coupled with a Thermo Flash EA 1112 elemental analyzer (Thermo, Bremen, Germany) at the Center for Physical Sciences and Technology in Vilnius, Lithuania. These compositions were calibrated relative to the VPDB and AIR scales using the following standards: USGS24, IAEA-CH3, IAEA-N1, IAEA-N2, and IAEA-600. Based on repeated measurements of the calibration standards and sample replicates, the precision (u(Rw)) was determined to be ±0.11‰ for δ^13^C and ±0.14‰ for δ^15^N. The total analytical uncertainty was estimated to be ±0.17‰ for δ^13^C and ±0.19‰ for δ^15^N.

### 2.4. Data Analysis

Before conducting data analyses, we tested the normality of the δ^13^C and δ^15^N distribution using the Shapiro–Wilk test (W). Both parameters conformed to a normal distribution (for δ^13^C: W = 0.96, *p* = 0.15; for δ^15^N: W = 0.98, *p* = 0.42). Therefore, we used parametric methods for subsequent analyses. Outliers were not present in δ^15^N; the single outlier in δ^13^C = −25.54‰ (Grubbs test, G = 3.14, *p* = 0.044) was not excluded from analysis.

To assess variation in stable isotope values, we first calculated the basic descriptive statistics (mean, standard deviation, and range) for δ^13^C and δ^15^N.

We examined species-level differences in δ^13^C and δ^15^N using a one-way ANOVA with species as the grouping factor, and then conducted pairwise t-tests to identify specific contrasts when appropriate. Habitat effects were tested using a one-way ANOVA with δ^13^C and δ^15^N as the dependent variables and habitat type (forest, wetland, or settlement) as the predictor variable. Post hoc pairwise t-tests were conducted where relevant. To examine potential intraspecific variation, separate ANOVAs were performed within each species to test for sex- and age-related differences in isotopic values.

The significance level was set at *p* < 0.05. Results with *p*-values between 0.05 and 0.10 were reported as non-significant trends. These results were interpreted descriptively using effect sizes and confidence intervals, rather than being considered evidence of statistical differences.

Using the SIBER framework [43], implemented in the SIBER package in R, we quantified isotopic niche width and overlap following A.L. Jackson et al. [44]. For each species, we calculated the standard ellipse area (SEA), which represents the core 40% of the bivariate isotope distribution and is analogous to one standard deviation in univariate space. To reduce bias from small samples, the small-sample-corrected ellipse area (SEAc) was computed using the (n − 1)/(n − 2) correction factor as recommended in [44]. These metrics provide comparable, sample-size-independent estimates of isotopic niche width.

To account for sampling uncertainty, we ran the Bayesian ellipse model in SIBER using the default weak priors (uninformative normal priors on the means and an inverse-Wishart prior for the covariance matrix) and generated 10,000 posterior draws for each species. SIBER calculates the Bayesian standard ellipse area (SEA.B) for each posterior sample as π√det(Σ), where Σ is the posterior covariance matrix. Posterior distributions were used to derive and report posterior means with 95% confidence intervals for isotopic niche size.

Pairwise niche overlap was estimated using SIBER functions that calculate proportional overlap between ellipses. These functions are based on (1) maximum-likelihood ellipses (SEA and SEAc) and (2) Bayesian posterior ellipses (SEA.B). This yields both point estimates and credible intervals. To visualize the total realized niche space, we also extracted the convex hull areas (TA), recognizing their sensitivity to sample size, yet retaining them for comparison with previous isotopic niche studies.

Since prey-specific isotope baselines were unavailable for the study area, the analyses were conducted using a relative isotopic framework. The results were interpreted in terms of trophic niche structure rather than quantitative diet composition.

Normality and outlier tests were performed in PAST version 4.13 (https://www.nhm.uio.no/english/research/resources/past/, accessed on 20 January 2025) Isotopic biplots were drawn in SigmaPlot ver. 12.5 (Systat Software Inc., San Jose, CA, USA), calculations were performed in Statistica for Windows, version 6.0 (StatSoft, Inc., Tulsa, OK, USA). All SIBER analyses, including SEA, SEAc, SEA.B, and ellipse overlap computations, were performed in SIBER v. 2.1.9. [43].

## 3. Results

### 3.1. Basic δ^13^C and δ^15^N Statistics

The stable isotope values differed among the analyzed mustelid species (Table 1). *M. putorius* exhibited intermediate δ^13^C values, while *M. foina* exhibited the highest values and *M. martes* exhibited the most depleted signatures. Differences were also evident for δ^15^N: *M. putorius* exhibited the highest mean δ^15^N, *M. foina* showed intermediate values, and *M. martes* had the lowest mean δ^15^N. The individual-level stable isotope dataset supporting this study is provided as Table S2, including species identity and δ^13^C and δ^15^N values for all analyzed individuals.

**Figure 2:**
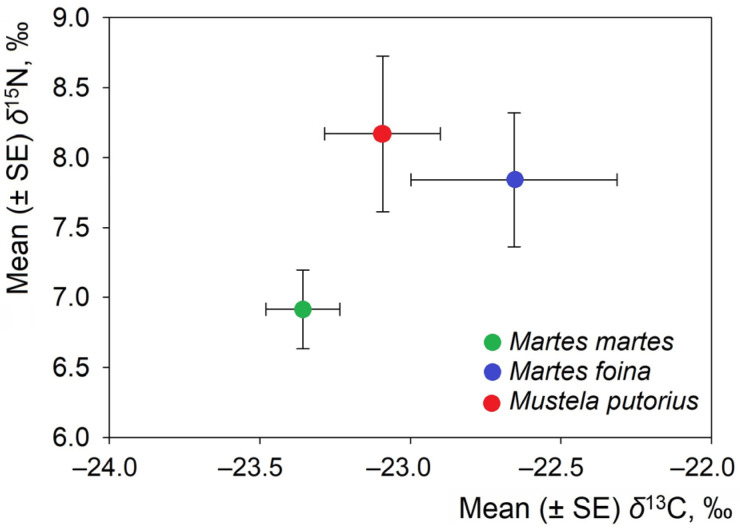
Position of *Martes martes*, *Martes foina*, and *Mustela putorius* in isotopic space based on mean (±SE) δ^13^C and δ^15^N values.

The relative positions of the investigated species in isotopic space (Figure 2) suggest a separation of species. The mean δ^13^C values were most depleted in *M.* *martes* (−23.35‰; 95% CI: −23.60 to −23.10‰), intermediate in *M. putorius* (−23.09‰; 95% CI: −23.54 to −22.63‰), and highest in *M. foina* (−22.65‰; 95% CI: −23.41 to −21.89‰). While these confidence intervals show partial separation between *M. foina* and *M. martes*, there was overlap among species overall. One-way ANOVA did not detect statistically significant differences in mean δ^13^C values (F = 2.92, *p* = 0.064).

The mean δ^15^N values were lowest in *M. martes* (6.94‰; 95% CI: 6.37–7.51‰), intermediate in *M. foina* (7.87‰; 95% CI: 6.81–8.92‰), and highest in *M. putorius* (8.20‰; 95% CI: 6.88–9.51‰). Confidence intervals overlapped among all three species, and differences in mean δ^15^N values were not statistically significant (ANOVA: F = 2.63, *p* = 0.083). Nevertheless, species identity accounted for a non-trivial proportion of variance in both isotope ratios (δ^13^C: SS_(effect) = 2.98, SS_(error) = 22.91; δ^15^N: SS_(effect) = 12.92, SS_(error) = 110.54), indicating directional differences among species means.

In terms of central isotopic tendencies, *M. martes* clusters toward lower δ^15^N and more depleted δ^13^C values. *M. foina* occupies an intermediate position, while *M. putorius* groups at the highest δ^15^N levels (Figure 2). Pairwise t-tests revealed the most significant difference in δ^13^C between *M. foina* and *M. martes*, with values of −22.65‰ and −23.35‰, respectively. This difference was statistically significant (t = 2.28, df = 38, *p* = 0.028). The largest difference in mean δ^15^N was observed between *M. putorius* (8.20‰) and *M. martes* (6.94‰). However, this difference was not statistically significant (t = 1.98, df = 39, *p* = 0.055). Nonetheless, the comparison represents the greatest nitrogen-based separation among the species pairs in terms of effect magnitude. Therefore, differences in mean isotope values are interpreted descriptively. The primary inference is based on isotopic niche structure rather than on marginal univariate *p*-values.

### 3.2. Species-Habitat Associations and Habitat Effect

**Figure 3:**
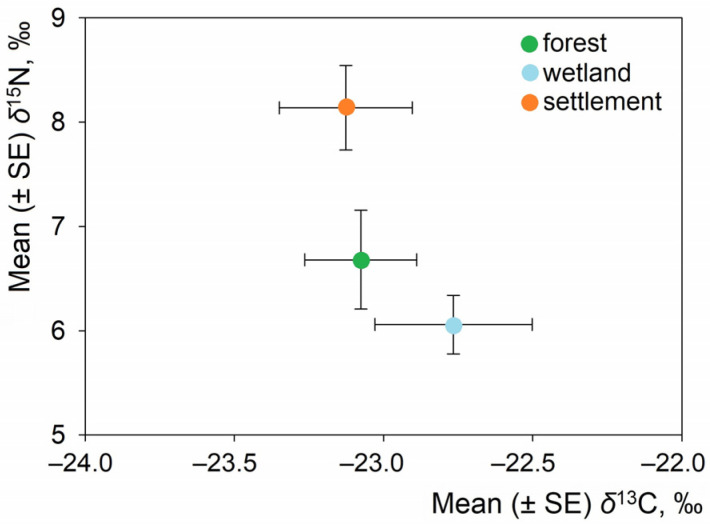
Position of mustelids (species pooled) in isotopic space based on mean (±SE) δ^13^C and δ^15^N values across habitats (forest, n = 13; wetland, n = 5; settlement, n = 17).

The analyzed mustelid species exhibited strong, albeit uneven, associations with different habitat types. *M. martes* was the only species found in all three habitat types, mostly in forests and occasionally in settlements and wetlands. *M. foina* were exclusively associated with settlements. *M. putorius* occurred mainly in settlements, with only one individual recorded in a forest habitat and none in wetlands. Therefore, the habitat analysis includes species-specific contrasts in isotopic values reflecting the three mustelids’ distinct habitat distributions (Figure 3).

When data from all species were pooled, the δ^13^C values revealed minimal variation among habitats. The mean δ^13^C value was −23.13‰ in settlements (95% CI: −23.58 to −22.67‰), −23.08‰ in forests (95% CI: −23.49 to −22.67‰), and −22.77‰ in wetlands (95% CI: −23.60 to −21.93‰). The confidence intervals overlapped broadly among the habitats, and the differences were not statistically significant (ANOVA: F = 0.33, *p* = 0.720). In contrast, δ^15^N values differed among habitats. The highest mean δ^15^N value was observed in individuals from settlements (8.14‰; 95% CI: 7.31–8.97‰), followed by individuals from forests (6.68‰; 95% CI: 5.65–7.72‰) and individuals from wetlands (6.06‰; 95% CI: 5.17–6.95‰). The effect of habitat on δ^15^N was statistically significant (ANOVA: F = 4.43, *p* = 0.020).

Pairwise comparisons supported these patterns. On average, individuals from settlements had higher δ^15^N values than those from forests (mean difference = 1.46‰; 95% CI: 0.15–2.77‰; t = 2.34, *p* = 0.026) and wetlands (mean difference = 2.08‰; 95% CI: 0.27–3.89‰; t = 2.42, *p* = 0.026). However, there was no significant difference in δ^15^N values between forest and wetland habitats (mean difference = 0.62‰; 95% CI: −1.21 to 2.45‰; t = −0.70, *p* = 0.494). For δ^13^C, none of the pairwise habitat comparisons were significant, and confidence intervals overlapped in all cases.

### 3.3. Intraspecific Aspects

**Figure 4:**
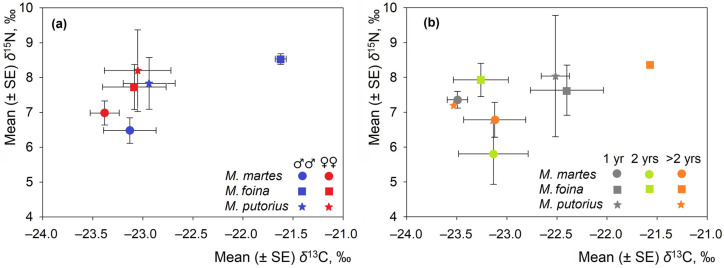
Mean (±SE) stable isotope values of δ^13^C and δ^15^N, plotted by (**a**) sex and (**b**) age class. Sample sizes are presented in Table S1.

There were no significant intraspecific differences in the isotopic positions of the mustelid species (Figure 4). The isotopic positions of males and females of *M. martes* and *M. putorius* are broadly similar, with overlapping standard error bars for both δ^13^C and δ^15^N within each species. The results of the ANOVA did not reveal any significant trends for *M. martes* (δ^13^C: F = 0.51, *p* = 0.48; δ^15^N: F = 0.39, *p* = 0.54) or *M. putorius* (δ^13^C and δ^15^N: F = 0.08, *p* = 0.79). In *M. foina*, though, δ^13^C in males was significantly higher (F = 7.20, *p* < 0.05), while δ^15^N did not differ significantly (F = 0.74, *p* = 0.43).

As shown in Figure 4b, individuals of different ages display partially overlapping δ^13^C and δ^15^N values within species, and no age class forms a distinct cluster. Overall, Figure 4 shows that neither sex nor age significantly alters the central isotopic tendencies of the species under investigation.

### 3.4. Isotopic Niche Size and Overlap

The isotopic niche size varied among the three mustelids. Standard Ellipse Areas (SEAs) were similar across species, with slightly higher values for *M. martes* (SEA = 3.23‰^2^) than for *M. foina* (SEA = 2.53‰^2^) and *M. putorius* (SEA = 2.68‰^2^). After applying a small-sample correction (SEAc), the isotopic niche widths were comparable across species, ranging from 3.04 to 3.33‰^2^ (Table 2). Bayesian ellipse areas (SEA.B) produced the same pattern: *M. martes* had the largest posterior niche area (mean SEA.B = 3.05‰^2^, 95% CI = 2.19–4.20‰^2^), followed by *M. putorius* (mean SEA.B = 2.06‰^2^, CI = 1.09–3.90‰^2^) and *M. foina* (SEA.B = 1.86‰^2^, CI = 0.97–3.52‰^2^).

**Figure 5:**
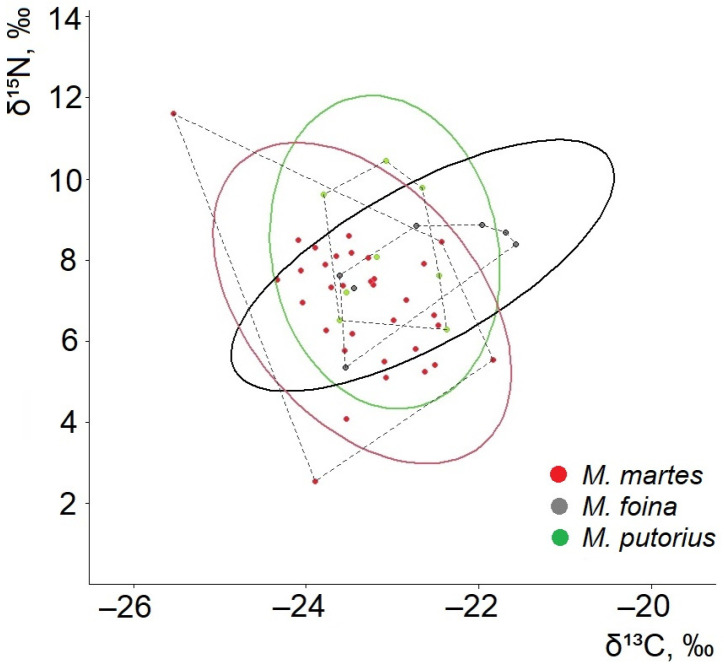
SIBER biplot of δ^15^N and δ^13^C values (‰). Each point represents an individual sample, and the colors indicate the mustelid species. The solid ellipses depict the SEAs, which represent the core isotopic niche for each species. The dashed polygons show the convex hulls that encompass the total niche width.

Trophic niche overlap analyses (Figure 5) revealed high trophic similarity between *M. foina* and *M. putorius*. Specifically, 57.4% of the *M. foina* SEA overlapped with that of *M. putorius*, and there was 54.2% overlap in the reverse direction. There was moderate overlap between *M. foina* and *M. martes*, comprising 23.2% of the *M. foina* SEA and 18.2% of the *M. martes* SEA. However, *M. martes* and *M. putorius* showed complete separation of isotopic niches (0% overlap). Convex hull areas (TA) indicated a substantially broader total isotopic space for *M. martes* (15.40‰^2^) compared to *M. foina* (3.47‰^2^) and *M. putorius* (4.37‰^2^), with the latter two species largely nested within the wider isotopic niche of the *M. martes* (Figure 5).

To evaluate the impact of unequal sample sizes, we performed a subsampling analysis: *M. martes* was repeatedly subsampled to align with the sample sizes of *M. foina* (n = 7) and *M. putorius* (n = 8). Subsampled *M. martes* consistently exhibited a broader isotopic niche than the other two species. When subsampled to n = 7, the mean ellipse area of *M. martes* was 2.59‰^2^ (SD = 1.56); when subsampled to n = 8, the mean ellipse area was 2.68‰^2^ (SD = 1.35). The relative ranking of species by niche width remained consistent, suggesting that differences in niche width are not an artifact of larger sample sizes but rather reflect greater isotopic dispersion.

### 3.5. Comparison of Contemporary and Historical Isotopic Positions

**Figure 6:**
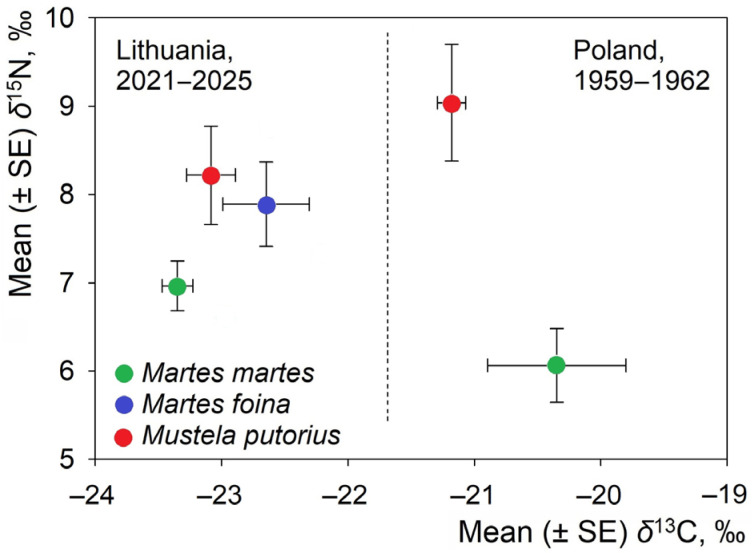
Comparison of position of sympatric mustelids in isotopic space in two countries and different time periods. Sample size in Poland: *M. martes*, n = 6, *M. putorius*, n = 14.

Stable isotope values of mustelids from contemporary Lithuania (2021–2025) differed markedly from those reported for historical specimens collected in Poland between 1959 and 1962 (Figure 6). Contemporary individuals of all three species occupied a relatively narrow range of δ^13^C values, whereas historical specimens showed substantially higher δ^13^C signatures.

For *M. martes*, historical samples exhibited significantly higher δ^13^C values than contemporary Lithuanian samples (t = 8.25, *p* < 0.0001). A similar pattern was observed for *M. putorius*, with historical specimens showing significantly higher δ^13^C values than contemporary individuals (t = 9.35, *p* < 0.0001). In contrast, δ^15^N values showed less pronounced differences between periods. Historical *M. putorius* exhibited slightly higher δ^15^N values than contemporary individuals, but this difference was not statistically significant (t = 1.2, *p* = 0.24).

In contemporary Lithuania, species were primarily differentiated along the δ^15^N axis, with *M. martes* occupying lower δ^15^N values, *M. foina* intermediate values, and *M. putorius* the highest values. The isotopic positions of the three species were closely clustered in δ^13^C space. In the historical Polish dataset, *M. martes* and *M. putorius* were more widely separated in isotopic space, particularly along the δ^13^C axis.

## 4. Discussion

Our study is the first to provide an isotopic comparison of assimilated diets in three sympatric mustelids. We fill a clear methodological gap by using δ^13^C and δ^15^N values from hair, which is a metabolically inert tissue suitable for integrating diet over a long time [36,37].

So far, most knowledge of trophic segregation in *M. martes* and *M. foina* comes from morphological diet analyses [45,46], seasonal diet studies [9,10,13], and habitat-dependent feeding patterns [47]. Similarly, the feeding ecology of *M. putorius* is primarily understood from conventional analyses describing vertebrate- and amphibian-dominated diets with geographic variation [21,22,23]. None of these investigations have used stable isotope analysis to directly compare their diets, relying on scat or stomach-content analysis [45,46], nutritional ecology syntheses [47], or spatial and habitat-use studies [3,48,49]. While these studies provide valuable context, highlighting differences in prey use, frugivory, and exploitation of anthropogenic resources, they cannot quantify assimilated diets or long-term trophic position.

### 4.1. Trophic Niche Width and Species Differences

Our results showed that *M. martes* occupied the broadest isotopic niche, which is consistent with the species’ well-documented ecological flexibility in forested landscapes. These findings are consistent with earlier studies demonstrating the species’ highly diverse diet, which includes small mammals, birds, fruits, and carrion [6,7,10,32,46,50,51]. A broader niche width likely reflects increased prey diversity in forest interiors and variability among individuals that exploit different food types, particularly small mammals and seasonally abundant fruits, across various European habitats [51,52].

Considerably narrower SEAs and more clustered isotopic values were exhibited by *M. foina* and *M. putorius.* This pattern is consistent with the trophic tendencies of both species. *M. foina* relies heavily on fruits, invertebrates, and synanthropic prey in settlements [12,15]. This strong association with human-modified landscapes, as well as the corresponding dietary flexibility, is supported by research indicating a significant consumption of plant foods, beetles, and human-related resources in rural and village settings [53,54,55].

*M. putorius* has a more strictly vertebrate-based diet [21,22], with a strong reliance on small mammals and other vertebrates [25,56]. The δ^13^C pattern further supports this. *M. foina* displayed the highest δ^13^C values, indicating increased use of resources linked to human environments.

The δ^13^C pattern further supports this. *M. foina* displayed the highest δ^13^C values, indicating an increased reliance on resources associated with human environments. This pattern is consistent with its documented synanthropic behavior and its frequent presence in orchards, villages, and agricultural structures [52,57].

### 4.2. Trophic Overlap and Mechanisms of Coexistence

Isotopic niche metrics revealed strong trophic convergence between *M. foina* and *M. putorius*. This high degree of overlap is consistent with previous findings indicating that both species rely more heavily on synanthropic rodents and other human-associated prey in agricultural or settlement environments [24]. The fact that both species were sampled almost exclusively in settlements in our dataset further supports this interpretation.

In contrast, the moderate overlap between *M. foina* and *M. martes* suggests that, although both consume small mammals and fruits, *M. foina* incorporates more human-related foods into its diet, while *M. martes* depends more strongly on forest prey. The near-zero overlap between *M. martes* and *M. putorius* highlights strong trophic segregation, likely due to habitat differences (forest versus settlement) and distinct prey preferences (rodents and birds versus rodents and amphibians). This pattern aligns with earlier suggestions of limited competition between *M. martes* and *M. putorius* [25], but our isotopic evidence quantifies these differences more precisely.

The convex-hull results showed that the narrower isotopic niches of *M. foina* and *M. putorius* were nested within the much larger isotopic space of *M. martes.* This indicates that *M. martes*’s diet encompasses a richer and more diverse prey landscape.

### 4.3. Influence of Habitat, Sex, and Age on Isotopic Signatures

Habitat was associated with variation in nitrogen values: individuals from settlements showed higher δ^15^N values, consistent with the use of higher-trophic-level or human-associated prey. This finding aligns with species ecology and habitat distribution in other countries. *M. foina* and *M. putorius* exploit synanthropic, rodent-rich prey communities in settlements, whereas *M. martes* is mainly found in forests [3,12,16,58]. These habitat associations are supported by studies showing that *M. foina* frequently occupy and maintain home ranges within human-dominated areas [58]. In contrast, *M. martes* have larger forest-based ranges and exhibit distinct spatial behavior linked to their preference for wooded habitats [59].

There were no substantial isotopic differences within species based on sex or age. With the exception of a minor difference in δ^13^C values in *M. foina*, males and females, as well as juveniles and adults, exhibited similar δ^13^C and δ^15^N values. These results suggest that interspecific ecological differences far outweigh intraspecific variation.

For example, although *M. putorius* exhibits pronounced sexual size dimorphism, their diets overlap extensively, with only minor and often statistically insignificant differences between males and females [60]. Similarly, sexual dimorphism in *M. putorius* does not translate into different prey choices [61]. He concluded that both sexes respond similarly to environmental prey availability rather than partitioning resources trophically. Even when slight seasonal differences were detected, previous studies have shown that prey abundance and habitat structure, rather than sex or age, are the dominant drivers of mustelid diets [21,61].

Several studies from different parts of Europe have shown that trophic divergence in martens related to sex or age is generally limited. Winter diet analyses of *M. foina* in Bulgaria revealed high dietary overlap between the sexes, with rodents dominating both diets, with trophic-niche breadth and Pianka’s index indicating strong overlap between the sexes [62]. For *M. martes*, some studies detected modest differences between the sexes, such as males consuming more ungulate carcasses or plant material and females consuming more squirrels or birds. However, these shifts varied across seasons and years, and were inconsistent in direction and magnitude, therefore Zalewski concluded that sexual size dimorphism does not produce stable dietary partitioning [63]. Similarly, age effects appear weak. In Swedish *M. martes*, it was found that juveniles and adults had similar prey compositions and body conditions, with only minor differences in consumption patterns during years with low microtine populations [64]. This indicates that resource availability, not age class, shapes dietary variation. Taken together, these studies reinforce the pattern observed in our isotopic data: intraspecific dietary differences linked to sex or age are subtle, context-dependent, and overshadowed by broader, habitat- and species-level ecological differentiation. Limited or inconsistent sexual trophic differences in *M. martes* are also confirmed by Monakhov [65].

### 4.4. Historical vs. Contemporary Isotopic Baselines

Prior to this study, the only reference for the isotopic niche of European mustelids was Selva et al. [34], which was based on 21 museum specimens collected from the pristine Białowieża Forest between 1959 and 1962. Selva et al. corrected the historical δ^13^C values for the Suess effect, suggesting that much of the long-term decline is due to changes in the atmospheric baseline rather than trophic shifts. Contemporary samples were collected over a short period, during which Suess-related change is negligible. Still, there were substantial differences not only in time period and habitat type but possibly also in prey baselines and anthropogenic influence. Nevertheless, the historical dataset is still useful as a reference. Mid-20th-century isotopic signatures represent non- or low-anthropogenic systems, while our contemporary results originate from more human-modified landscapes. The current strong isotopic convergence of *M. foina* and *M. putorius* may reflect increased synanthropy and homogenized prey communities in modern rural–urban mosaics. Therefore, a comparison of our dataset with Selva’s emphasizes both species- and time-based analyses (Figure 6). Figure 6 illustrates the differences in the isotopic positions of mustelids in present-day Lithuania (2021–2025) relative to historical records from Poland (1959–1962). In Lithuania, all three species cluster within a relatively narrow δ^13^C range, reflecting similar carbon sources across the landscape. However, the species differ in δ^15^N, reflecting their characteristic dietary patterns. *M. martes* relies more on forest prey and plant material, *M. foina* makes greater use of mixed and synanthropic food resources, and *M. putorius* shows a stronger dependence on vertebrate-rich diets. The tight clustering and comparatively small error bars for the Lithuanian samples also indicate a relatively cohesive and contemporary environmental isotopic baseline.

In contrast, the historical Polish dataset exhibits markedly different isotopic positions. *M. martes* and *M. putorius* from the 1959–1962 samples have substantially higher δ^13^C values, which reflect a different environmental baseline likely related to pristine forest conditions and the lower incorporation of resources linked to human environments at that time. *M. putorius* also has higher δ^15^N values than Lithuanian *M. putorius*, and they are more distinct from *M. martes* than they are today. These findings suggest a more distinct trophic structure in mid-20th-century forest ecosystems, possibly due to higher prey specialization, reduced synanthropy, and a more intact prey community.

Together, the two datasets illustrate a temporal shift in mustelid isotopic niches. Modern species in Lithuania exhibit stronger trophic convergence, particularly between *M. foina* and *M. putorius*, whereas historical Polish specimens demonstrate clearer dietary segregation and significantly different baseline isotopic values. The observed convergence may be a reflection of increased synanthropy and homogenized prey communities in modern human-modified landscapes. Following Hobson [35], it also reinforces the importance of modern isotopic studies for understanding contemporary mesocarnivore ecology.

The differences between historical and contemporary isotopic signatures are consistent with potential long-term ecological or anthropogenic changes; however, they cannot be attributed to specific causes without baseline correction. Therefore, these patterns represent hypotheses rather than confirmed effects. Because prey-specific baselines were not available, these temporal differences cannot be causally attributed to anthropogenic change and should be interpreted as hypotheses.

### 4.5. Human-Related Drivers of Trophic and Niche Convergence

Analyses of carnivore responses to human-modified landscapes [52,56,66] suggest that isotopic shifts are part of a broader pattern in which anthropogenic disturbances compress trophic and ecological niches, promoting convergence among formerly segregated mesocarnivores. P.J. Manlick and J.N. Pauli demonstrated [67] that human disturbance increases δ^15^N and δ^13^C values, widens isotopic niches, and increases dietary overlap among martens, foxes, and coyotes across North America. Similar trends have been reported in fine-scale diet studies. For example, in Californian open-space preserves, human activity reduced dietary partitioning among coyotes, bobcats, and foxes, primarily through increased nocturnality and reliance on shared, human-associated prey [68]. These patterns parallel our finding of stronger trophic convergence in modern Lithuanian mustelids and suggest that human-altered prey communities homogenize carnivore foraging niches.

At broader spatial scales, mesocarnivores also respond consistently to anthropogenic gradients. In Mediterranean systems, stone martens, foxes, and genets increasingly use woodland and cropland patches proximate to humans. These habitats provide refuge and human-related food resources regardless of reduced availability of natural prey [69]. This spatial attraction mirrors isotopic evidence of subsidized foraging, indicating that habitat restructuring and food subsidies jointly drive niche compression. Overall, human-dominated landscapes may favor generalist strategies and reduced dietary specialization, as suggested by isotopic and dietary patterns reported across mesocarnivore systems [70,71,72]. Intensification of land use also promotes temporal niche convergence, with mesocarnivores shifting toward shared nocturnal activity under high human influence [73,74].

### 4.6. Study Limitations

Interpretation of niche width and overlap metrics for *M. foina* and *M. putorius* is constrained by small and uneven sample sizes. Although Bayesian approaches partially account for this limitation, niche size and overlap estimates for these two species should be considered preliminary. Likewise, the lack of significant sex- or age-related isotopic differences should not be interpreted as evidence that such factors are unimportant; rather, the study likely lacked sufficient power to detect subtle intraspecific variation. Future studies with larger, more balanced samples are required to confirm the observed patterns and to robustly evaluate intraspecific niche differentiation. The strong association of *M. foina* and *M. putorius* with settlements in our sample further reduces our ability to distinguish species effects from habitat effects. These limitations do not invalidate the detected interspecific patterns; however, conclusions should be interpreted cautiously and viewed as pattern-based hypotheses requiring confirmation with larger, seasonally balanced datasets.

Seasonality and hair growth can influence isotopic interpretations. In our dataset, individuals were collected over the course of many months; however, monthly sample sizes (Table S1) were too small to test for seasonal differences. Consequently, the isotopic signatures should be viewed as broad, time-averaged dietary indicators rather than season-specific values. Subtle seasonal shifts in prey use may therefore remain undetected.

Stable isotope analysis integrates diet from all consumed food sources, but it does not allow for the partitioning of prey by taxon or the quantification of their proportional contributions. Additionally, since prey-specific isotopic baselines were not analyzed, interspecific and habitat-related differences should be interpreted as integrated trophic patterns rather than precise dietary reconstructions. Therefore, the results identify patterns that warrant further investigation using larger, habitat-balanced datasets.

## 5. Conclusions

Our study is the first to provide a contemporary isotopic comparison of three mustelid species in Northern Europe. Despite their partial spatial coexistence, their isotopic signatures suggest moderate dietary differentiation. The clearest separation is reflected in isotopic niche metrics rather than in traditional significance tests of mean δ^13^C and δ^15^N values.

*M. martes* had the broadest isotopic niche, which is consistent with its diverse, forest-based diet. *M. foina* and *M. putorius* had narrower isotopic niches with substantial overlap. This pattern is supported by SIBER ellipse analyses and is consistent with their shared occurrence in human settlements. However, because species and habitat were not evenly represented across sampling locations, these patterns should be interpreted cautiously and cannot be attributed to habitat effects alone.

The isotopic niche of *M. martes* had minimal overlap with that of *M. putorius* and moderate overlap with *M. foina*. Negligible sex- and age-related differences in δ^13^C and δ^15^N indicate that interspecific ecological differences exceed intraspecific variation within species.

Overall, our findings demonstrate the value of isotopic approaches for assessing trophic relationships among mustelids, showing that species primarily differ in isotopic niche structure rather than in mean isotope values.

## Figures and Tables

**Table 1:** Central position (mean ± SD) and ranges of stable isotope ratios in the hair of mustelids.

Species	n	Mean δ^13^C (‰) ± SD	Range (Min–Max)	Mean δ^15^N (‰) ± SD	Range (Min–Max)
*Martes martes*	33	−23.35 ± 0.71	−25.54–−21.84	6.94 ± 1.62	2.55–11.62
*Martes foina*	8	−22.65 ± 0.91	−23.62–−21.58	7.87 ±1.26	5.35–8.86
*Mustela putorius*	7	−23.09 ± 0.54	−23.80–−22.38	8.20 ± 1.58	6.29–10.46

**Table 2:** Summary of core isotopic niche metrics generated by SIBER, illustrating differences in dietary niche breadth among mustelid species.

Species	SEA (‰^2^) ^1^	SEAc (‰^2^)
*Martes foina*	2.53	3.04
*Martes martes*	3.23	3.33
*Mustela putorius*	2.68	3.12

^1^ SEA—Standard Ellipse Area; SEAc—Standard Ellipse Area corrected for small sample size.

## Data Availability

We are ready to share raw data on the basis of scientific cooperation.

## Supplementary Materials

The following supporting information can be downloaded at: https://www.mdpi.com/article/10.3390/life16020208/s1, Table S1: Sample size of European pine marten (*Martes martes*), stone marten (*M. foina*) and European polecat (*Mustela putorius*) from Lithuania, with breakdown by species, year, month, sex, and age (available not for all individuals); Table S2: Individual-level stable isotope values of mustelids.

## Abbreviations

The following abbreviations are used in this manuscript:

SIBER	Stable Isotope Bayesian Ellipses in R
SEA	Standard Ellipse Area
SEAc	Standard Ellipse Area corrected for small sample size
SEA.B	Bayesian Ellipse Area
