# Supplementary material for: Isotopic Niche of Three Sympatric Mustelids
Source: Life (Basel). 2026 Jan 27;16(2):208. doi: 10.3390/life16020208 (PMC12942384; doi:10.3390/life16020208)
Supplement: Supplementary file 1 [file life-16-00208-s001.zip › life-4113366-supplementary.pdf]

life

MDPI

## Supplements

**Table S1**. Sample size of European pine marten (*Martes martes*), stone marten (*M. foina*), and European polecat (*Mustela putorius*) from Lithuania, with breakdown by species, year, month, sex, and age (available not for all individuals).

| Year/month/sex/age | *Martes martes* | *Martes foina* | *Mustela putorius* |
|---|---|---|---|
| 2021 | 1 | | |
| 2023 | 6 | 1 | 1 |
| 2024 | 13 | 4 | 4 |
| 2025 | 8 | 2 | 5 |
| January | 2 | 1 | |
| February | 8 | 1 | 1 |
| March | 2 | 1 | 2 |
| April | 8 | 2 | 1 |
| June | 1 | | |
| October | 1 | | 2 |
| November | 1 | | |
| December | 5 | 2 | 1 |
| Male | 5 | 3 | 4 |
| Female | 26 | 5 | 3 |
| Sex unknown | 2 | 0 | 0 |
| 1 year | 14 | 3 | 2 |
| 2 years | 6 | 3 | |
| > 2 years | 6 | 2 | 1 |
| Age unknown | 7 | 0 | 4 |

**Table S2.** Individual-level stable isotope values of mustelids.

| Species | $\delta^{13}C$ (‰) | $\delta^{15}N$ (‰) |
|---|---|---|
| *Martes martes* | –24.06 | 7.74 |
| *Martes martes* | –23.90 | 2.55 |
| *Martes martes* | –23.48 | 8.19 |
| *Martes martes* | –23.65 | 8.10 |
| *Martes martes* | –23.72 | 7.33 |
| *Martes martes* | –23.57 | 7.38 |
| *Martes martes* | –23.21 | 7.53 |
| *Martes martes* | –23.22 | 7.39 |
| *Martes martes* | –23.28 | 8.06 |
| *Martes martes* | –24.09 | 8.49 |
| *Martes martes* | –23.90 | 8.30 |
| *Martes martes* | –24.05 | 6.96 |
| *Martes martes* | –23.77 | 6.28 |
| *Martes martes* | –23.47 | 6.19 |
| *Martes martes* | –23.78 | 7.89 |
| *Martes martes* | –22.44 | 8.46 |
| *Martes martes* | –23.51 | 8.60 |
| *Martes martes* | –24.33 | 7.51 |

| | | |
|---|---|---|
| *Martes martes* | –22.99 | 6.52 |
| *Martes martes* | –25.54 | 11.62 |
| *Martes martes* | –22.74 | 5.81 |
| *Martes martes* | –23.08 | 5.11 |
| *Martes martes* | –23.25 | 7.48 |
| *Martes martes* | –22.63 | 5.25 |
| *Martes martes* | –22.51 | 5.42 |
| *Martes martes* | –23.54 | 4.09 |
| *Martes martes* | –23.09 | 5.51 |
| *Martes martes* | –22.63 | 7.91 |
| *Martes martes* | –23.56 | 5.78 |
| *Martes martes* | –22.48 | 6.39 |
| *Martes martes* | –22.52 | 6.64 |
| *Martes martes* | –22.84 | 7.02 |
| *Martes martes* | –21.84 | 5.53 |
| *Martes foina* | –23.62 | 7.63 |
| *Martes foina* | –23.45 | 7.30 |
| *Martes foina* | –23.55 | 5.35 |
| *Martes foina* | –21.69 | 8.69 |
| *Martes foina* | –21.58 | 8.38 |
| *Martes foina* | –22.72 | 8.86 |
| *Martes foina* | –21.96 | 8.86 |
| *Mustela putorius* | –23.08 | 10.46 |
| *Mustela putorius* | –23.61 | 6.53 |
| *Mustela putorius* | –23.53 | 7.20 |
| *Mustela putorius* | –23.80 | 9.62 |
| *Mustela putorius* | –22.46 | 7.62 |
| *Mustela putorius* | –23.19 | 8.08 |
| *Mustela putorius* | –22.66 | 9.78 |
| *Mustela putorius* | –22.38 | 6.29 |

https://doi.org/10.3390/xxxxx
